# Quality indicators in the endoscopic detection of gastric cancer

**DOI:** 10.1002/deo2.221

**Published:** 2023-04-09

**Authors:** Vikneswaran Namasivayam, Noriya Uedo

**Affiliations:** ^1^ Department of Gastroenterology and Hepatology Singapore General Hospital Singapore; ^2^ Department of Gastrointestinal Oncology Osaka International Cancer Institute Osaka Japan

**Keywords:** diagnosis, early cancer detection, endoscopy, gastric cancer, quality

## Abstract

Gastroscopy is the reference standard for the diagnosis of gastric cancer, but it is operator‐dependent and associated with missed gastric cancer. The proliferation of gastroscopic examinations, increasingly for the screening and detection of subtle premalignant lesions, has motivated scrutiny of quality in gastroscopy. The concept of a high‐quality endoscopic examination for the detection of superficial gastric neoplasia has been defined by expert guidelines to improve mucosal visualization, engender a systematic examination process and detect superficial neoplasia. This review discusses the evidence supporting the components of a high‐quality diagnostic gastroscopic examination in relation to the detection of gastric cancer, and their potential role as procedural quality indicators to drive a structured improvement in clinically meaningful outcomes.

## INTRODUCTION

Gastroscopy (EGD) is the reference standard for the diagnosis of gastric cancer (GC). Current detection of GC is premised on EGD as the final common diagnostic pathway, either as a primary screening or confirmatory modality in high‐incidence countries, a surveillance modality in intermediate‐risk population, or as a diagnostic modality.^1^, ^2^, ^3^ EGD has a high negative predictive value with interval GCs diagnosed in less than 1% following a normal examination.^4^, ^5^

Yet 10% of all GCs had a negative endoscopic examination within the preceding three years.^4^ While GC that was not detected on initial EGD has been attributed to a complex interplay of multiple factors, including missed lesions, inadequate follow‐up of lesions, inadequate surveillance of premalignant conditions, and de novo cancer development, GC may be missed during endoscopy. A similar phenomenon has been observed in studies of patients with negative EGD followed over time,^4^ comparisons of preoperative endoscopic findings with gastrectomy specimens in patients with GC,^6^, ^7^, ^8^ and in studies evaluating the proportion of missed synchronous lesions in patients undergoing ESD.^9^, ^10^ Missed cancers are also associated with shorter survival.^11^

The risk of missed cancers, in turn, has been associated with factors that may be linked to the technical performance of EGD. Missed cancers have been associated with small tumor size,^12^, ^13^, ^14^ flat or depressed morphology,^13^, ^15^ gastric body location,^4^, ^8^, ^13^, ^16^ posterior wall location,^8^ endoscopists’ inexperience,^17^, ^18^ EGD performed at primary care,^19^ endoscopy lists with greater numbers of procedures^20^ and an inadequate number of biopsies^4^ and shorter observation times.^21^

The impetus for high‐quality gastroscopic examination is derived from the following trends. Advances in endoscopic interventions for early GC have shifted the focus of endoscopic examinations towards the detection of superficial gastric neoplasia which are subtle and easily missed. Recent guidelines advocating surveillance endoscopies for gastric intestinal metaplasia – and the increased procedural volume and cost that would ensue – are predicated on the detection of these subtle lesions.^1^, ^3^ While image‐enhanced endoscopy to aid the detection and characterization of superficial neoplasia has been endorsed and adopted into mainstream practice, advances in imaging modalities and endoscopy systems risk rendering the endoscopist the weak link in this man‐machine interface. These developments, in addition to revolutionizing the diagnosis of early GC, have heralded a philosophical shift in the goals of a diagnostic gastroscopic examination‐ from being a purely diagnostic test to rule in or rule out a clinical diagnosis in a Bayesian manner to regarding every diagnostic EGD as an opportunity to screen for GC.^22^ These secular trends underscore a need for gastroscopic evaluation to be optimized, on a consistent basis, to detect early gastric neoplasia.

To this end, the concept of a high‐quality endoscopic examination for the detection of superficial gastric neoplasia has been defined by expert guidelines to improve mucosal visualization, engender a systematic examination process and recognize and diagnose superficial neoplasia without compromising subsequent endoscopic resection. Yet EGD is an operator‐dependent procedure where endoscopic performance is variable, and detection of abnormalities entails a learning curve. Variations have been observed in the detection rates of neoplasia in upper gastrointestinal endoscopic examinations.^23^

The experience with colonoscopy indicates that there is variation in the effective execution of most aspects of colorectal cancer screening and surveillance and the implementation of auditable measures has led to a significant improvement in quality. The growing demand for EGDs performed for detecting premalignant lesions has motivated a similar scrutiny of quality in EGD.

Efforts to improve the quality of endoscopy are based on formulating a framework of metrics that serve as benchmarks for measuring and improving the quality of healthcare. These parameters, termed quality indicators, serve as specific goals to direct quality improvement efforts and are usually reported as the proportion of correct performance or as the proportion of interventions that achieve a predefined goal.^24^ Quality indicators consist of three categories: structural measures assess a healthcare system's capacity to provide care; process measures assess the delivery of care; and outcome measures assess the results of care. These measures pertain to the pre‐procedure, intra‐procedure, and post‐procedure timeframes.^25^

The ideal quality indicator is measurable, strongly associated with a relevant clinical outcome (e.g., interval cancer), demonstrates a wide variation in endoscopist performance, and is amenable to improvement with interventions.^21^ In colonoscopy, this is most closely exemplified by the adenoma detection rate, which, despite its limitations, has served as a validated metric to drive quality improvement. Adenoma detection rate is associated with the risk of interval colorectal cancer and fatal interval colorectal cancers.^26^, ^27^, ^28^ It demonstrates a wide variation – up to 10‐fold – between endoscopists with high and low detection rates. Improvements in adenoma detection rate have been associated with reduced risk of interval colorectal cancer and fatal interval colorectal cancer, highlighting its utility in monitoring response to remediation efforts.^29^

The implementation of quality in upper endoscopy entails several challenges. Unlike colonoscopy which examines a single organ and is performed mostly for colorectal cancer screening and surveillance, upper endoscopy examines three different organs and is performed for a diverse range of indications. It is a common modality for the evaluation and treatment of a diverse range of cancers that vary in their epidemiology, endoscopic appearance, and the populations at risk. Furthermore, upper endoscopy has evolved from being a procedure to being a platform for a diverse range of procedures that encompass cancer treatment, third‐space endoscopy, and bariatric indications. Hence it is not feasible to encapsulate the quality of an upper endoscopic examination with a single summary measure. The challenge lies in finding and adopting quality measures that are appropriate for the indication of the procedure and are associated with improved clinical outcomes while avoiding a proliferation of measures. This review appraises the evidence supporting the current recommendation for a high‐quality diagnostic examination and their potential candidacy as quality indicators as they relate to GC detection and does not include therapeutic endoscopic procedures (see Table 1).

**TABLE 1:** The components of a high‐quality examination

Components of a high‐quality examination	Take home messages
Gastric mucosal visualization	A validated global assessment metric for the adequacy of gastric mucosal visualization is lacking Premedication with N‐acetylcysteine and simethicone improves mucosal visualization but has not been shown to improve the detection of gastric neoplasia. Antispasmodics reduces peristalsis but has not been shown to improve the detection of gastric neoplasia. The impact of fasting on neoplasia detection needs to be clarified. Recommendations on fasting relate to the risks of sedation and aspiration rather than neoplasia detection.
Photo documentation	Systematic photo documentation is recommended as proof of a complete examination. Systematic screening protocol of 22 gastric images has been recommended. The optimal number of images has not been determined.
Examination time	A shorter examination time is associated with a lower detection rate of gastric neoplasia. The optimal examination time and the minimum threshold examination time have not been defined.
Sedation	Current recommendations on sedation primarily focus on safety and aspiration risk. Observational data suggest sedation may improve the detection of neoplasia.
Endoscopist biopsy rate (EBR)	EBR is associated with the detection of gastric precancerous lesions. Prospective, outcome‐based studies are needed to validate its use as a quality metric.
Image‐enhanced endoscopy	IEE improves the detection of atrophic and metaplastic mucosa and characterization of gastric neoplasms.
Artificial intelligence	AI may reduce the miss rate for gastric neoplasms. AI may reduce blind spots and increase inspection times during upper endoscopy AI m ay potentially improve detection, delineation of neoplastic margins, and depth of invasion though more studies are needed.

## RATES OF GC DETECTION AND MISSED GC

To date, there are no validated quality indicators for EGD that are associated with the risk of interval GC. While preventing interval GCs is the goal of improving quality in gastroscopic examinations, the rate of missed GC per se is not suitable as a quality indicator. Generally defined as the portion of GCs that had a negative EGD in the preceding 3 years, missed GC rate does not measure the proportion of endoscopic examinations that missed a GC. Also, it does not measure the performance of an individual endoscopist. Similarly, the GC detection rate on screening endoscopy is an unsuitable outcome measure for quality improvement. In a Korean population with a high GC incidence, the GC detection rate was 2.61 per 1000 screening.^30^ According to the annual report of the Japan Society of Gastrointestinal Cancer Screening in 2018, the GC detection rate in Japan is 1.66 per 1000.^29^ Even in high GC incidence countries, the rates are too low to be a feasible measure of quality as a large number of procedures would be required to obtain a precise measurement with a narrow confidence interval. The GC detection rates would be expected to vary with baseline cancer risk in heterogeneous populations and would be arguably even lower in populations with lower GC incidence.

## MUCOSAL VISUALIZATION

The importance of adequate gastric mucosal visualization in detecting subtle lesions is now well recognized and forms the basis of recommendations for adequate gaseous luminal distension and rinsing of mucus and bubbles during EGD. Unlike colonoscopy which routinely scores the quality of bowel preparation, a validated global assessment metric for the adequacy of gastric mucosal visualization is lacking. Instead, the quality of mucosal visualization is largely addressed by surrogate measures including premedication and fasting.

The use of premedication to improve mucosal visualization encompasses mucolytics and deforming agents and antispasmodic medication. Premedication with N‐acetylcysteine and simethicone improves mucosal visualization but has not been demonstrated to translate into an increase in the detection of gastric neoplasia.^31^, ^32^, ^33^, ^34^, ^35^ The use of antispasmodics is a useful adjunct in reducing peristalsis that may obscure gastric views but the evidence on improving the detection of neoplasms is limited and conflicting.^36^, ^37^ While fasting is a prerequisite for an endoscopic examination, expert recommendations on fasting relate to the risks of sedation and aspiration rather than the adequacy of mucosal visualization for cancer detection.^25^, ^38^

While these specific measures contribute to a high‐quality examination, their salutary effects could be subsumed under a more general measure of gastric mucosal visualization which may be more amenable to quality improvement. In this regard, the use of scoring systems for mucosal visibility that grades the extent of mucus has largely been used in the research setting and has not found traction in clinical practice.^39^

## PHOTO DOCUMENTATION

Image documentation has been long recognized as an integral component of a complete endoscopic examination.^40^ It serves as a legal record of a complete examination, consciously addresses endoscopic blind spots, serves as a reference in endoscopic serial examinations and lesions referred for endoscopic resection, and facilitates audit and teaching.

While accepted as proof of a complete examination, the role of systematic photo‐documentation in improving the detection of early cancers requires further elucidation. The optimal number of photos to detect cancers has not been ascertained. Guidelines differ in the minimum number of photos that should be taken and their specific locations, often reflecting epidemiological variations in the GI cancers of interest. The spectrum ranges from no stipulation of a minimum number of photos by some societies,^41^, ^42^ a minimum of 10 photos^43^, ^44^ to 22 gastric images.^45^ The systematic screening protocol comprising 22 gastric images was developed as a standard to avoid blind spots and has gained traction but has yet to be universally adopted.^45^ The photo documentation of the major papilla has been associated with the detection of upper GI neoplasms and is regarded as a surrogate for the endoscopist's proficiency and attention.^46^ The use of an alphanumeric code system has been described which entails photo documenting unique areas and registering the sequence of the procedure by numbering the areas. This may have value in engendering a common framework for endoluminal orientation but it requires further study.^47^ The increasing use of chromoendoscopy and image‐enhanced endoscopy may also require the capture of additional images and requires further clarification.

The need for a comprehensive image record of an examination must be balanced against the real‐world limitations in image processing and archival infrastructure. While photo documentation has become an indispensable feature of a high‐quality endoscopic examination, the current evidence for specifying a minimum number of photos as a universal quality indicator is limited.

## EXAMINATION TIME

Multiple observation studies have demonstrated that a longer examination time is associated with increased detection of gastric neoplasia.^48^, ^49^, ^50^ In addition, increasing examination time by implementing an institutional protocol of a minimum examination time has been shown to increase the detection of upper GI neoplasia.^20^ However, studies have varied in their definition of examination time – from intubation to extubation of the patient, from time of withdrawal from D2 to extubation, inclusive or exclusive of biopsies – as well as the cutoffs used for categorizing fast and slow endoscopists. The studies are observational with residual confounding by variables relevant to GC such as *H* *elicobacter* *pylori* infection. In addition, these studies relate to the average examination time and not the duration of any given individual examination. Lengthening examination times may also increase the need for sedation with implications for practice settings where routine sedation is not practiced. Existing expert guidelines, while acknowledging the importance of adequate examination time, and recommending its documentation at least for surveillance procedures, have mostly refrained from specifying a minimum examination time.^41^, ^42^, ^43^, ^51^, ^52^ The minimum threshold examination time needs to be defined as there are practical constraints to the time that may be devoted to endoscopic examination. Future studies should determine the effect of specific cutoff examination times on the detection rates to determine the optimal examination time and if there is a ceiling effect beyond which no benefit is achieved in detection.

## SEDATION

Existing recommendations on sedation largely address safety and aspiration risks from upper endoscopy including the levels of sedation that can be delivered by endoscopists, risk assessment, the degree of cardiorespiratory monitoring and the team pause to ensure the correct procedure is being performed on the correct patient.^41^, ^42^ While sedation has been recommended to enable the detection of neoplasia on the grounds that it facilitates adequate examination time and improves patients’ acceptance and willingness to undergo repeat endoscopies, the evidence is limited.^51^, ^53^ In a multicenter retrospective study of over 430,000 endoscopies, sedation was associated with increased detection of early cancer and high‐grade intra‐epithelial neoplasia, probably by facilitating a longer examination, the use of accessory techniques and sufficient time for biopsies.^54^ Sedation practices, including the sedation regimen and the specific procedures that require sedation, also vary widely and these reflect differences in licensing requirements, healthcare policies, norms, and remuneration.^55^ Sedation is also associated with cardiopulmonary risks.^56^ Robust studies are needed to clarify the effect of sedation on neoplasia detection to guide and influence any widespread change in practice.

## ENDOSCOPIST BIOPSY RATE

The endoscopist biopsy rate (EBR), defined as the percentage of EGD with at least one biopsy sampling for histology from the esophagus, stomach, or duodenum, exclusive of biopsies for rapid urease testing has recently attracted attention as a potential quality metric. In a multi‐centered retrospective cohort study including almost 30 000 outpatient EGDs, the EBR was strongly associated with the detection of gastric premalignant conditions (GPC) – defined as atrophic gastritis, intestinal metaplasia, and dysplasia – as well as the risk of missed GC. The rates of EBR also demonstrated wide variability among endoscopists, ranging from 22% to 66%. The improved sensitivity with EBR was at the expense of a higher rate of negative biopsies which were associated inversely with EBR.^57^ EBR is easy to calculate as it only requires the number of EGD with at least one biopsy for histology from any part of the upper gastrointestinal tract and the total number of EGD. However, most of the endoscopies in this study were performed using standard video‐resolution endoscopes, a significant minority underwent multiple endoscopic examinations, the study did not address the confounding effect of image‐enhanced endoscopy or the examination time and EBR did not discriminate between lesional and non‐lesional biopsies. Absolute EBR values probably reflect differences in the baseline risks of cancer and are likely to vary in practice settings. EBR increases costs related to negative biopsies and is also susceptible to gaming (“one biopsy and done”). EBR also brings to the fore a fundamental difference in competing philosophies of diagnostic endoscopy ‐ those that favor a biopsy‐based strategy and those that increasingly prioritize meticulous inspection and characterization with the aid of advanced imaging modalities while minimizing biopsies. Further prospective studies are needed in diverse practice settings with varying baseline risks to clarify the role of EBR.

## *HELICOBACTER PYLORI* INFECTION STATUS

**FIGURE 1:**
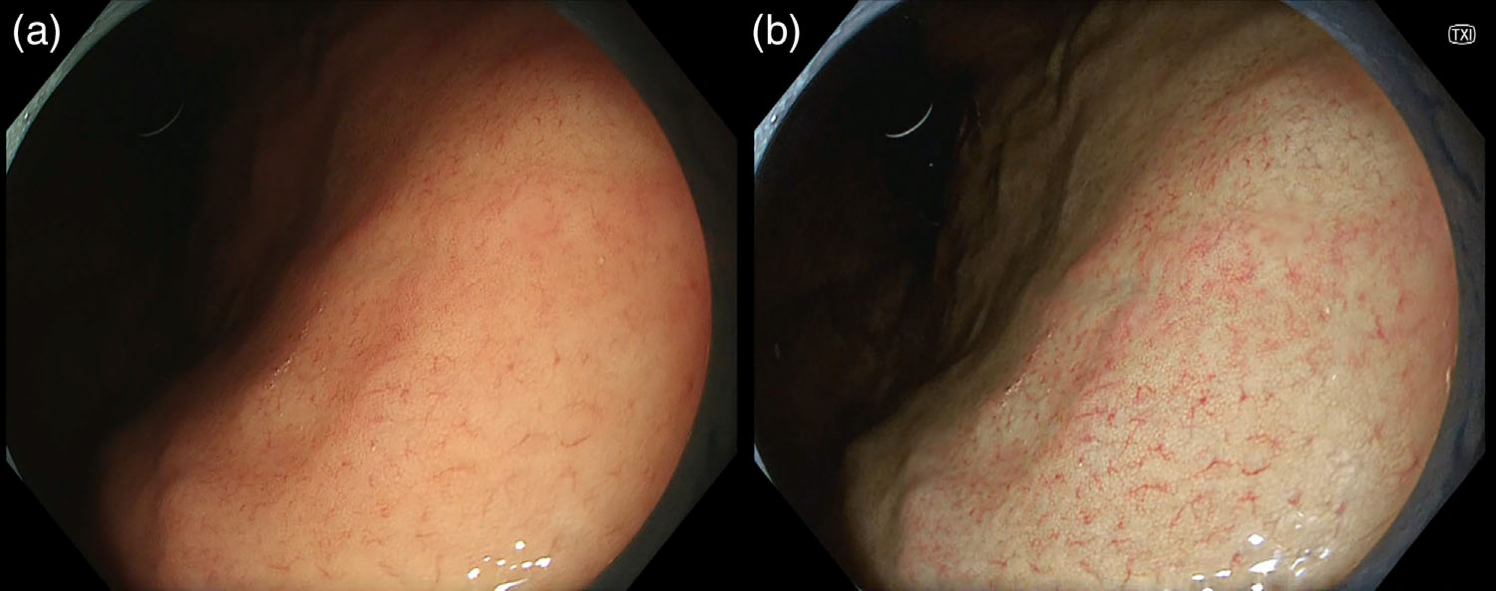
Regular arrangement of collecting venule is seen in the lesser curvature of the stomach, indicating *Helicobacter pylori* infection naïve status (a). The finding is enhanced in texture and color enhancement mode (b).

**FIGURE 2:**
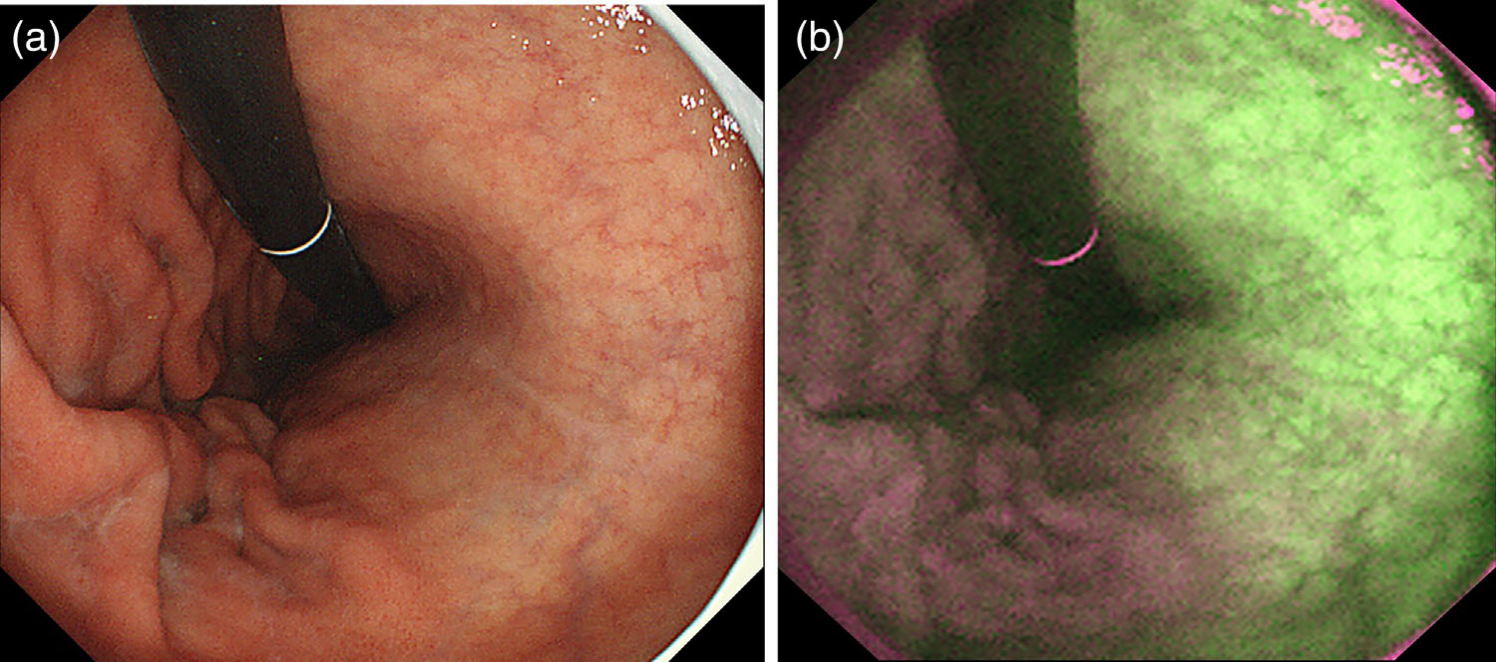
Endoscopic atrophy characterized by the increased visibility of the vessels, pale mucosal color, and low mucosal height is seen in the lesser curvature side of the gastric corpus (a). This finding indicates current or past *Helicobacter pylori* infection. The finding is enhanced in autofluorescence mode (b).

**FIGURE 3:**
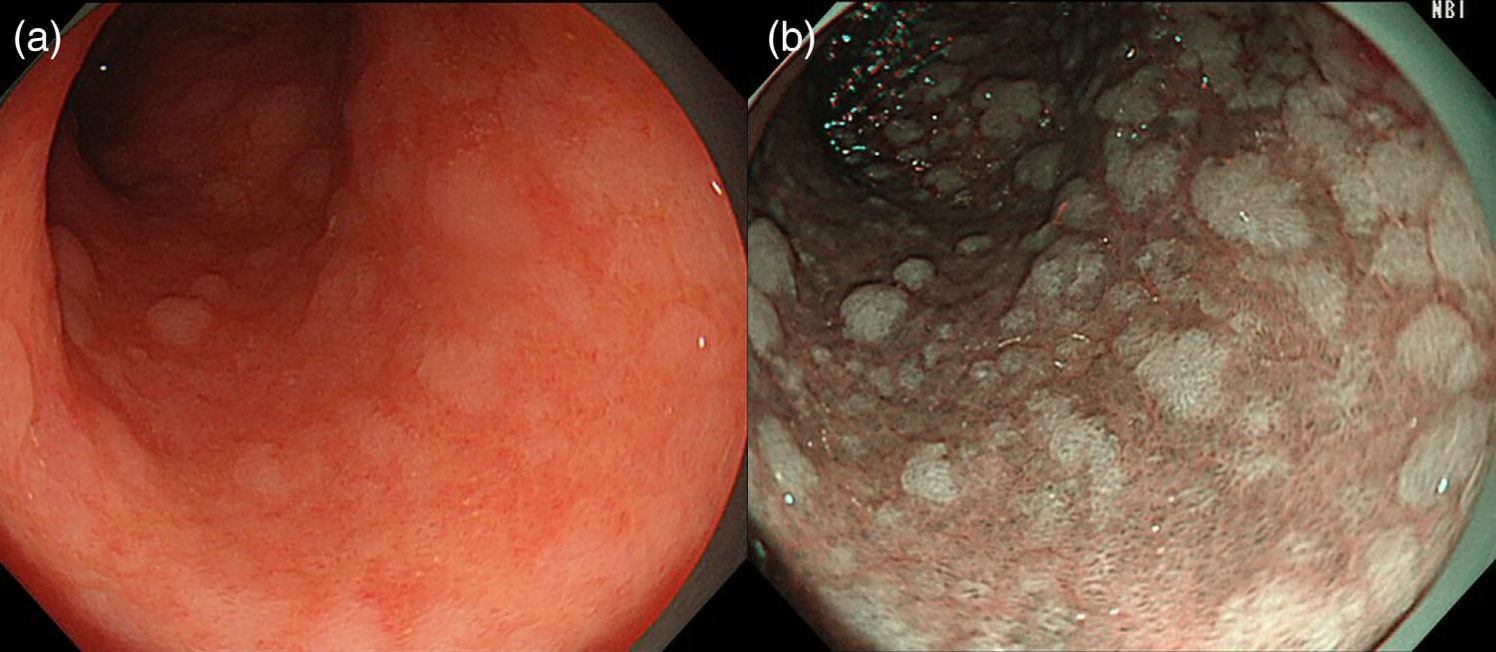
Endoscopic intestinal metaplasia in the antrum as whitish elevated lesions (a). This finding indicates current or past *Helicobacter pylori* infection. The finding is enhanced in narrow‐band imaging mode (b).

**FIGURE 4:**
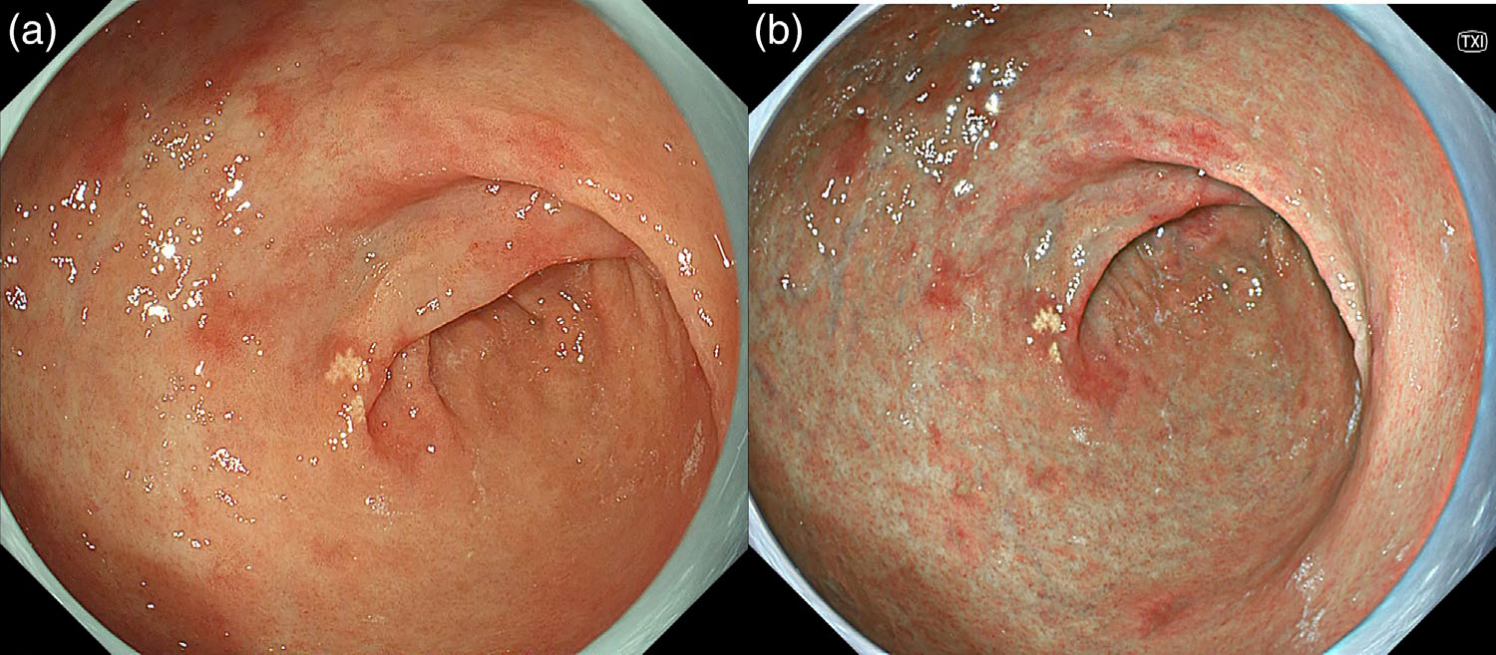
Endoscopic intestinal metaplasia in the antrum as reddish depressed lesions (a). A small xanthoma is seen in the center of the image. These findings suggest past *Helicobacter pylori* infection. The finding is enhanced in texture and color enhancement mode (b).

The real‐time endoscopic diagnosis of *H*. *pylori* infection has gained interest for the following reasons. *H. pylori* is a class I carcinogen that causes GC.^58^ In *H. pylori* infection naïve patients, GC risk is very low,^59^ while *H. pylori* infection increases GC risk up to 6‐fold.^60^ Although *H. pylori* eradication halves the risk of GC (relative risk = 0.54; 95% confidence interval [CI] 0.40–0.72), there still remains a residual risk of GC that is higher than *H. pylori* infection naïve patients.^61^ Therefore tests for *H. pylori* infection may underestimate the risk of GC in patients with past infection of *H. pylori* that had been eradicated. Hence the real‐time endoscopic diagnosis of *H. pylori* infection status identifies an at‐risk stomach that should prompt careful inspection for neoplasia. Several endoscopic findings of gastric mucosa have been reported to be associated with *H. pylori* infection (Figures 1, 2, 3, 4).^62^ While these findings are useful in identifying an infected stomach that may be at increased risk of GC, the role of endoscopic diagnosis of *H. pylori* infection as a quality metric for GC requires clarification.

## IMAGE‐ENHANCED ENDOSCOPY

**FIGURE 5:**
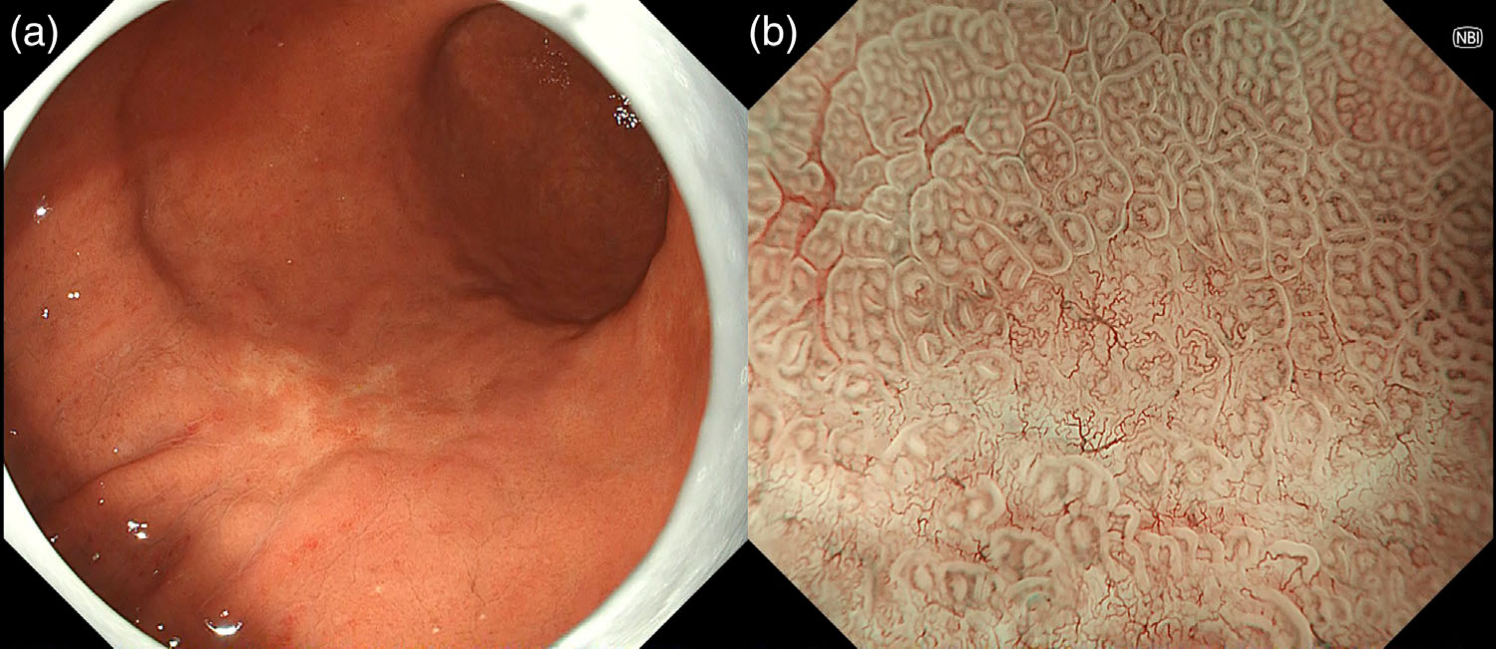
Superficial undifferentiated type early gastric cancer in the greater curvature of the gastric body (a). Irregular microvessels within the demarcation line are observed in the lesion ([b], white box in [a]).

The advent of image‐enhanced endoscopy (IEE) has addressed some of the shortcomings of white light examination in detecting mucosa at risk of GC and the detection and characterization of gastric neoplasia. IEE improves the detection of precancerous atrophic and metaplastic gastric mucosa and shows promise as a tool for mapping the severity of metaplastic change.^63^, ^64^, ^65^, ^66^, ^67^ IEE also enables endoscopic diagnosis of *H. pylori* infection status.^68^, ^69^ IEE, especially with magnification, aids in the characterization of gastric neoplasia but detection has been more challenging (Figure 5), A multi‐centered randomized controlled trial of over 4500 patients comparing second‐generation NBI with white light imaging showed no significant difference in the detection of early GC.^71^ NBI did have a better positive predictive value in diagnosing cancer. Furthermore, a one‐year follow‐up of this high‐risk cohort demonstrated that intensive index endoscopy, with both white light and second‐generation NBI, was not associated with a reduction in the rates of detections of new GC.^70^ Hence the limitations of both modalities highlight an unmet need in detecting early cancers. The application of IEE also involves a learning curve though this may be negotiated with training.^72^ While guidelines recommend the use of IEE, preferably with magnification, many endoscopy units may not have access to these technologies and functions and use standard resolution white light endoscopes and biopsy sampling as the surveillance and diagnostic strategy.

## ARTIFICIAL INTELLIGENCE

The application of artificial intelligence (AI) in endoscopy has great potential in augmenting quality improvement efforts by addressing human and systemic shortcomings that underpin variations in performance. Computer vision applications may address underperformance, AI may provide real‐time tracking and feedback on quality during endoscopy, serve as a training tool, automate abstraction from unstructured data for quality reporting, and potentially generate novel quality indicator metrics.

AI has been shown to reduce the miss rate for gastric neoplasms, evaluate the quality of endoscopic examination, improve quality by reducing blind spots, and increase inspection times during upper endoscopy.^73^, ^74^, ^75^, ^76^, ^77^ AI systems have been trained to improve the detection, delineation of neoplastic margins, and depth of invasion although more studies are needed on its real‐time performance.^78^, ^79^, ^80^ AI may potentially assist to improve the performance of inexperienced endoscopists through robust studies needed to support the clinical application.^81^ Natural language processing has been applied to abstract unstructured data to calculate and report quality metrics in colonoscopy and a similar application in EGD would simplify quality reporting by relieving the burden of calculating metrics.^82^, ^83^ However clinical trials with relevant outcome measures are needed in diverse patient groups and practice settings to realize the promise of AI.

## CONCLUSION

The real‐world practice of diagnostic EGD is imperfect and is associated with missed GC. The increasing application of EGD for the detection of superficial early neoplasia has been coupled with a drive to improve the quality of gastroscopic examinations. While the components of a high‐quality gastroscopic examination have been defined, there remains a dearth of evidence to support their role as formal quality indicators to drive a structured improvement in clinically meaningful outcomes. This is further complicated by the diversity of indications that upper endoscopy has evolved to address which may lead to a proliferation of quality measures. AI holds the promise of addressing variations in performance, but this must be validated in clinical trials with relevant outcomes in diverse patient groups and practice settings, before its adoption.

## CONFLICT OF INTEREST STATEMENT

None.
